# Botulinum Toxin in Aesthetic Medicine: A Bibliometric Analysis of Research Trends and Methodological Quality of the Top 100 Cited Publications

**DOI:** 10.1093/asjof/ojae131

**Published:** 2025-01-08

**Authors:** Zhen Yu Wong, Pegah Damavandi, Maksim Richards, Pojsakorn Danpanichkul, Oluwatobi Adegboye, Ryan Faderani, Muholan Kanapathy, Afshin Mosahebi

## Abstract

**Background:**

Botulinum toxin is widely used in aesthetic medicine, with numerous studies examining its efficacy and safety.

**Objectives:**

This bibliometric analysis aims to describe research trends and assess the methodological quality of the highest-impact botulinum toxin research in aesthetic applications.

**Methods:**

The authors of this study identified the 100 most-cited publications on botulinum toxin in aesthetics using Web of Science, covering all available journal years (from inception to October 2024). The Oxford Centre for Evidence-Based Medicine Level of Evidence (LOE) was used to assess the methodological quality of each study.

**Results:**

The authors identified 1728 articles on the aesthetic uses of botulinum toxin, with the top 100 most-cited articles spanning from 1994 to 2021. The United States dominated the research landscape with 50 articles, followed by Canada (15). The University of California (United States) and the University of British Columbia (Canada) emerged as the top contributing institutions. Among journals, *Dermatologic Surgery* led in publication count, followed by *Plastic and Reconstructive Surgery* and *Aesthetics Surgery Journal*. Notably, Professors Jean Carruthers and Alastair Carruthers from Canada were the leading researchers, topping both publication count and citation metrics. Notably, more than half of the studies were classified as LOE 5 (Expert Opinion/Narrative Review).

**Conclusions:**

This bibliometric analysis reveals a paucity of high-quality studies in the field of botulinum toxin in aesthetic medicine, with research predominantly concentrated in western countries. These findings highlight the need for more rigorous, evidence-based studies and increased global collaboration to advance the understanding and application of botulinum toxin in aesthetics.

**Level of Evidence: 4 (Therapeutic):**

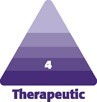

Botulinum toxin is a neurotoxic protein produced by the bacterium *Clostridium botulinum.*^1^ It temporarily paralyzes targeted muscles by inhibiting the release of acetylcholine at the neuromuscular junction.^2^ Although botulinum toxin has many therapeutic applications, for example, in treating chronic migraines, spasticity, and hyperhidrosis, its use in aesthetic medicine has become more widely recognized over the past 2 decades.^3^ Primarily employed to reduce the appearance of facial wrinkles and lines, because of its minimally invasive use, botulinum toxin has become a cornerstone of nonsurgical cosmetic procedures.^4^ The increasing demand for botulinum toxin in aesthetic treatments often overshadows the importance of understanding its safety and long-term effects, particularly because it continues to gain popularity and follow an upward pattern of use in cosmetic procedure trends.^5^

Bibliometric analysis is a valuable tool when attempting to gain a comprehensive understanding of botulinum toxin aesthetics. It involves quantitative evaluation of published literature, demonstrating research trends, influential studies, and potential gaps in knowledge.^6^ This method allows researchers to assess the scope of existing literature, identify key contributors in the field, and highlight areas where further investigation is needed.^7^ In this study, we aim to assess the existing literature on botulinum toxin in aesthetics to provide a detailed overview of current research trends and evaluate the quality of the highest-level research done for the topic, while highlighting a low level of evidence (LOE) in certain aspects of the field.

## METHODS

### Data Collection

On October 11, 2024, citation data related to aesthetic uses of the botulinum toxin were retrieved from the Web of Science Core Collection (WoSCC).^8^ The search strategy employed in the WoSCC online database included the following string: TI/AB = (“botulinum toxin” OR “neuro botulinum toxin” OR “botulinum neurotoxin” OR “BoNT-A” OR “botox” OR “dysport” OR “xeomin” OR “botulinum toxin” OR “botulinum neurotoxin” OR “daxxify” OR “relaxin” OR “purton” OR “jouveau” OR “letaba” OR “vistable” OR “vistabel” OR “aqualure” OR “biocouture” OR “onabotulinumtoxinA” OR “abobotulinumtoxinA” OR “incobotulinumtoxinA” OR “prabotulinumtoxin” OR “daxibotulinumtoxinA-lanm” OR “letibotulinumtoxin” OR “daxibotulinumtoxinA” OR “CNBTX-A” OR “CBTX A” OR “lifton” OR “redux” OR “chinatax” OR “rt001” OR “neurobot” OR “naboth” OR “botulae” OR “relator” OR “proscene” OR “prosigna” OR “medigoxin” OR “infobox” OR “lantos” OR “neurobot”) AND (“Skin” OR “Dermatology” OR “Dermatologic Surgical Procedures” OR “Dermatologic Surgical Procedure” OR “Procedure, Dermatologic Surgical” OR “Procedures, Dermatologic Surgical” OR “Surgical Procedure, Dermatologic” OR “Surgical Procedures, Dermatologic” OR “Cutaneous Surgical Procedures” OR “Cutaneous Surgical Procedure” OR “Procedure, Cutaneous Surgical” OR “Procedures, Cutaneous Surgical” OR “Surgical Procedure, Cutaneous” OR “Surgical Procedures, Cutaneous” OR “Skin Surgery” OR “Skin Surgeries” OR “Surgeries, Skin” OR “Surgery, Skin” OR “Cutaneous Surgery” OR “Cutaneous Surgeries” OR “Surgeries, Cutaneous” OR “Surgery, Cutaneous” OR “Dermatologic Surgery” OR “Dermatologic Surgeries” OR “Surgeries, Dermatologic” OR “Surgery, Plastic” OR “Plastic Surgery” OR “Esthetic Surgery” OR “Esthetic Surgeries” OR “Surgeries, Esthetic” OR “Surgery, Cosmetic” OR “Cosmetic Surgery” OR “Wound Healing” OR “Healing, Wound” OR “Healings, Wound” OR “Wound Healings” OR “Re-Epithelialization” OR “Reepithelialization” OR “Wound Epithelialization” OR “Epithelialization, Wound” OR “Aesthetic*”). No restrictions were placed on the time period or language of the publications.

Literature screening was conducted to eliminate duplicates and retracted articles, a process carried out independently by 2 researchers. The 100 most-cited publications on aesthetic uses of the botulinum toxin were identified. Any disagreements that arose during this screening were resolved by senior authors. For each publication, we collected the following basic data: title, publication year, country or region, institution, journal, references, and keywords. The Oxford Centre for Evidence-Based Medicine (OCEBM) LOE and prevalent themes for included articles were ascertained.^9^

### Bibliometric Analysis

The included articles were analyzed and visualized using Biblioshiny (R), VOSviewer (Leiden University, Leiden, Netherlands), and Bibliometrics Online Analysis Platform. The primary outcomes of interest included trends over time, study design, LOE, main research themes, authorship, affiliations, countries of origin, and total citations. Additionally, cluster analyses were performed to examine the relationships between nations or regions, institutions, journals, research categories, keywords, and references.

## RESULTS

**Figure 1.**
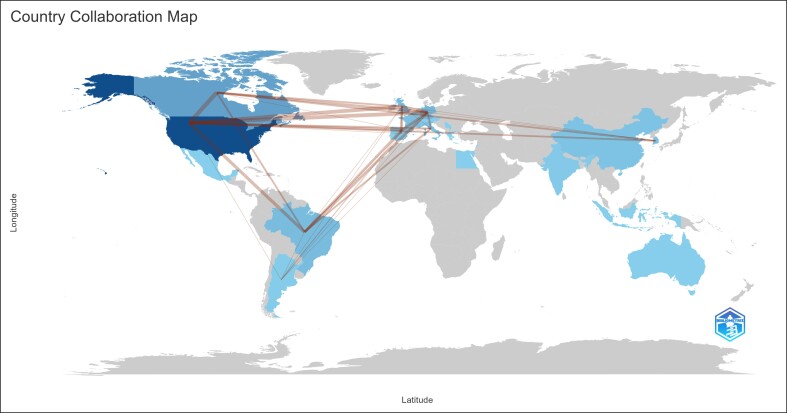
An adapted global map of contributing countries of the top 100 most-cited articles.

A comprehensive analysis of the literature on aesthetic uses of the botulinum toxin was conducted, identifying 1728 articles, with the top 100 cited articles spanning from 1994 to 2021 and originating from 23 countries (Figure 1 and Supplementary Table 1).^10-109^ The United States led with 50 articles, followed by Canada with 15, China with 5, France with 5, and South Korea with 4. Figure 1C illustrates the temporal distribution of the top 100 cited articles, categorized by year of publication.

Author affiliations revealed contributions from 124 institutions to the 100 highly cited articles. Table 1 lists the top 5 institutions by publication frequency, led by the University of California (United States) and the University of British Columbia (Canada). The articles were published in 23 journals, whereas *Dermatologic Surgery* led in publication count, followed by the *Plastic and Reconstructive Surgery* and *Aesthetics Surgery Journal*. Overall, 331 authors contributed to these articles, with J. Carruthers and A. Carruthers from Canada leading in publication count and citations.

**Table 1.** The Distribution of Publication Frequency of the Top 5 Most-Cited Institutions

Institutions	Article
University of California	57
University of British Columbia	27
University of North Carolina	12
University of Alabama	12
Berlin Institute of Health	10

Applying Bradford's Law, 1 source was in Zone 1, 4 sources were in Zone 2, and 18 sources were in Zone 3. The analysis also encompassed 5487 citations, averaging 54.87 citations per article. Most studies were classified as OCEBM LOE 5 (53%), followed by Level 1 (17%), Level 31 (16%), Level 2 (7%), and Level 4 (7%). In terms of study design, the majority were expert opinion/narrative reviews (53%), with smaller numbers of randomized controlled trials (17%), case–control studies (14%), and other study designs, including case series, systematic review, and post hoc analysis.

**Figure 2.**
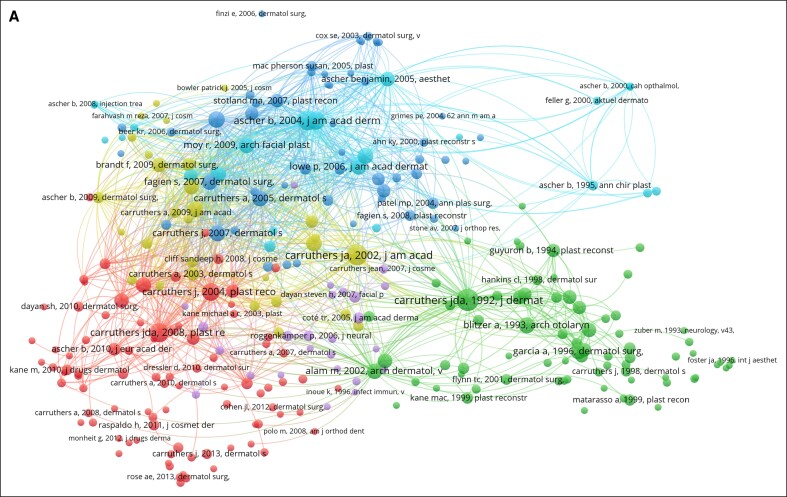

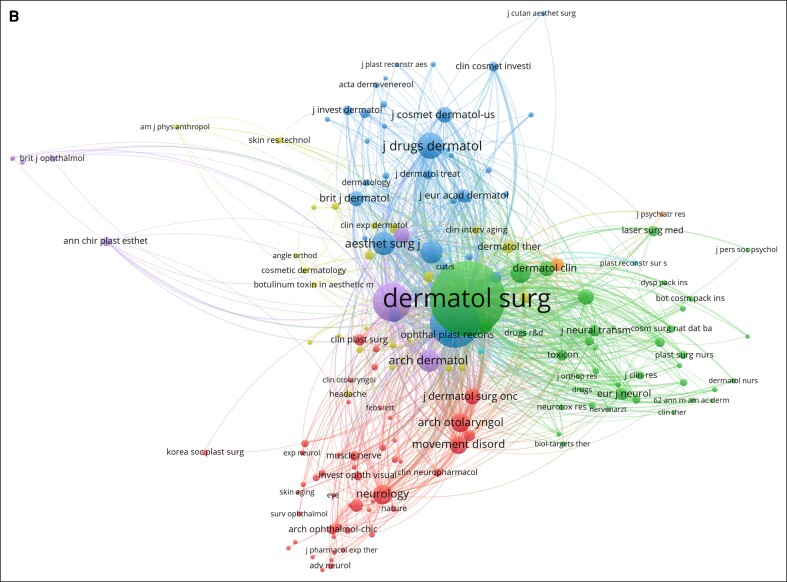

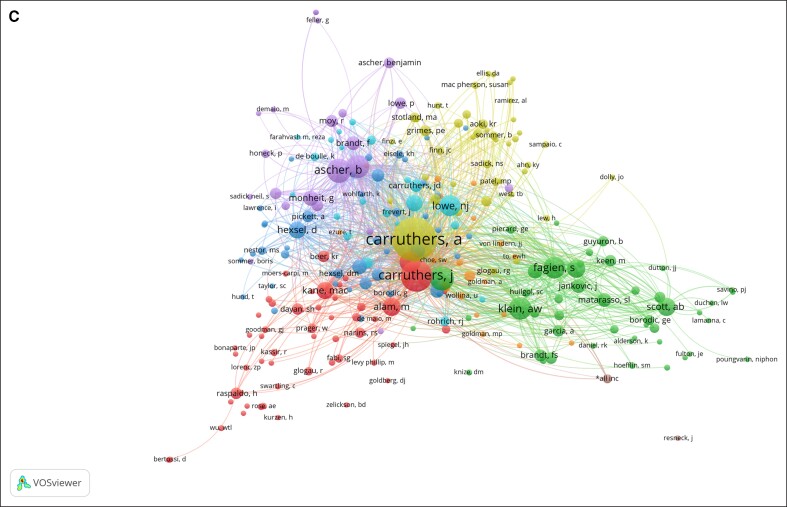
(A) The reference collaboration network of the top 100 most-cited articles. (B) The journal collaboration network of the top 100 most-cited articles. (C) The author collaboration network of the top 100 most-cited articles.

**Figure 3.**
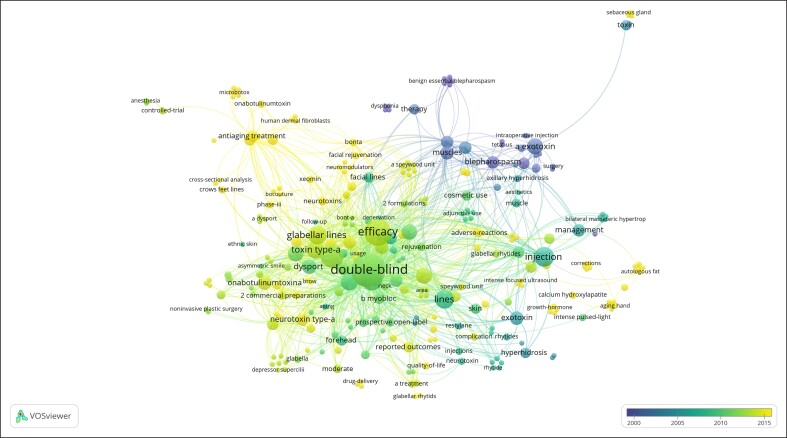
The keyword collaboration network of the top 100 most-cited articles.

For co-citation analysis (Figure 2A-C), the threshold of a minimum of 3 documents included 291 references, 161 journals, and 253 authors. To identify research hotspots and developmental trends in the aesthetic uses of the botulinum toxin, a co-occurrence cluster analysis based on keywords was performed using VOSviewer software. A total of 277 keywords were analyzed, with the resulting network maps displayed in Figure 3. The most frequently used keywords were “Double-blind,” “Efficacy,” “Safety,” and “Injection.”

## DISCUSSION

This study represents the first bibliometric analysis focused specifically on the use of botulinum toxin in aesthetic medicine, providing a comprehensive overview of the research landscape in this field. Our analysis revealed trends in geographical distribution of research, publishing journals, and authors producing the literature. Notably, the data indicated that the United States, Dermatologic Surgery, and J. Carruthers and A. Carruthers led in publication count and citations. This summary of the existing literature provides a foundation for future research and demonstrates the importance of continuing such research into the aesthetic applications of botulinum toxin.

Among the studies reviewed, the top 3 most-cited papers are particularly noteworthy. The first was authored by Professor J. Carruthers who is globally recognized for her work in both cosmetic surgery and procedures. A Canadian ophthalmologist by background, Dr J. Carruthers authored the top-cited paper, a consensus recommendation published in 2008. It explores how significant an in-depth knowledge of facial anatomy and aging is and how alongside a thorough understanding of properties and techniques, it can impact optimal outcomes in aesthetics.^110^ This paper has been cited 279 times, reflecting how highly both this specific research and author are valued in this field. The second most highly cited paper was again a consensus recommendation by Carruthers in 2004. It provides guidelines on issues such as treating the individual, specifics of the procedure regarding dosing and injection sites, and patient selection and counseling. A testament to its value in the field of botulinum toxin use, this paper has acquired 226 citations.^98^ The third paper was authored by Sundaram, a board-certified dermatologist with a special interest in the scientific basis of new aesthetic technologies.^111^ A group of dermatologists and plastic surgeons convened the Global Aesthetics Consensus Group to provide consensus recommendations for the aesthetic use of botulinum toxin Type A. The paper emphasized that to achieve desirable outcomes in populations that are rapidly changing, the use of botulinum toxin must have a patient-centered approach. This paper accumulated a citation count of 133. These studies have clearly influenced current understanding and practices in the use of botulinum toxin for aesthetics.

Making substantial contributions to the literature on botulinum toxin aesthetics, as previously mentioned, the most-cited authors with the highest publication count are, unsurprisingly, J. Carruthers and A. Carruthers. Their work, which spans 16 publications, has received a total of 1310 citations. One of the most significant studies they co-authored includes a paper published in 1992. This piece of literature pioneered the use of botulinum toxin for cosmetic purposes, the first paper on the cosmetic use of neuromodulators published in the *Journal of Dermatologic Surgery and Oncology*.^112^ The cumulation of their work demonstrates their invaluable role in acquiring knowledge in botulinum toxin aesthetics and cosmetic procedures in general.

Bibliometric analysis provides valuable insights into the research landscape; however, it does come with its limitations. One of these is that citation numbers, while indicative of influence, do not necessarily reflect the quality or clinical relevance of a study.^113^ Additionally, analysis may be skewed by literature more recently published that has not yet had time to accumulate citations, with citation itself being subject to the bias from the author.^114,115^ Furthermore, bibliometric data often favor English-language publications, potentially overlooking significant research in other languages.^116^ Because of such limitations, readers and researchers are advised to individually evaluate the efficiency of each method and its bearings on the results.

## CONCLUSIONS

Despite these limitations, this bibliometric analysis offers an in-depth overview of the literature available within the topic area, with research mainly published and cited from western countries. It clearly demonstrates the need for more evidence-based studies, particularly from differing areas of the globe, and identifies key areas for future research of botulinum toxin in aesthetics.

## Supplemental Material

This article contains supplemental material located online at https://doi.org/10.1093/asjof/ojae131.

## Supplementary Material

ojae131_Supplementary_Data
