# Supplementary material for: Botulinum Toxin in Aesthetic Medicine: A Bibliometric Analysis of Research Trends and Methodological Quality of the Top 100 Cited Publications
Source: Aesthet Surg J Open Forum. 2025 Jan 8;7:ojae131. doi: 10.1093/asjof/ojae131 (PMC11836436; doi:10.1093/asjof/ojae131)
Supplement: ojae131_Supplementary_Data [file ojae131_supplementary_data.docx]

| **Study** | **Journal** | **Study design** | **OCEBM Levels of Evidence** | **Total Citation** |
| --- | --- | --- | --- | --- |
| Carruthers 2008 | Plastic And Reconstructive Surgery | Expert Opinion / Narrative Review | 5 | 279 |
| Carruthers 2004 | Plastic And Reconstructive Surgery | Expert Opinion / Narrative Review | 5 | 226 |
| Sundaram 2016 | Plastic And Reconstructive Surgery | Expert Opinion / Narrative Review | 5 | 133 |
| Keen 1994 | Plastic And Reconstructive Surgery | Randomised controlled trial | 1 | 129 |
| Kim 2005 | Plastic And Reconstructive Surgery | Case series | 4 | 128 |
| Brandt 2009 | Dermatologic Surgery | Randomised controlled trial | 1 | 108 |
| Guyuron 1994 | Plastic And Reconstructive Surgery | Case control study | 3 | 106 |
| Carruthers 1998 | Dermatologic Surgery | Expert Opinion / Narrative Review | 5 | 102 |
| Fagien 1999 | Plastic And Reconstructive Surgery | Expert Opinion / Narrative Review | 5 | 101 |
| Kane 2009 | Plastic And Reconstructive Surgery | Randomised controlled trial | 1 | 97 |
| Carruthers 2010 | Dermatol Surg | Randomised controlled trial | 1 | 86 |
| Rzany 2007 | Dermatologic Surgery | Case control study | 3 | 85 |
| Mazzuco 2010 | J Am Acad Dermatol | Case control study | 3 | 85 |
| Small 2014 | American Family Physician | Expert Opinion / Narrative Review | 5 | 83 |
| Brandt 1998 | Dermatologic Surgery | Expert Opinion / Narrative Review | 5 | 83 |
| Carruthers 2003 | Dermatologic Surgery | Expert Opinion / Narrative Review | 5 | 82 |
| Yu 2007 | Aesthetic Plastic Surgery | Case control study | 3 | 79 |
| Ahn 2000 | Plastic And Reconstructive Surgery | Case series | 4 | 77 |
| Carruthers 2007 | Dermatologic Surgery | Randomised controlled trial | 1 | 75 |
| Frankel 1998 | Archives Of Otolaryngology-Head & Neck Surgery | Case control study | 3 | 72 |
| Ahn 2013 | Dermatologic Surgery | Expert Opinion / Narrative Review | 5 | 72 |
| Ascher 2010 | Journal Of The European Academy Of Dermatology And Venereology | Expert Opinion / Narrative Review | 5 | 72 |
| Ascher 2010 | Journal Of The European Academy Of Dermatology And Venereology | Expert Opinion / Narrative Review | 5 | 67 |
| Cavallini 2014 | Dermatologic Surgery | Systematic review | 2 | 66 |
| Lorenc 2013 | Aesthetic Plastic Surgery | Expert Opinion / Narrative Review | 5 | 65 |
| Binder 1998 | Dermatologic Surgery | Expert Opinion / Narrative Review | 5 | 65 |
| Fagien 2008 | Plastic And Reconstructive Surgery | Expert Opinion / Narrative Review | 5 | 64 |
| Lee | Dermatologic Surgery | Case series | 4 | 63 |
| Hankins 1998 | Dermatologic Surgery | Prospective cohort study | 2 | 62 |
| Wollina 2012 | American Journal Of Clinical Dermatology | Expert Opinion / Narrative Review | 5 | 61 |
| Flynn 2010 | American Journal Of Clinical Dermatology | Systematic review | 2 | 61 |
| Sommer 2003 | Dermatologic Surgery | Case control study | 3 | 59 |
| Carruthers 2016 | Dermatologic Surgery | Expert Opinion / Narrative Review | 5 | 59 |
| Kane 1999 | Plastic And Reconstructive Surgery | Case control study | 3 | 58 |
| Suber 2014 | Aesthet Surg J | Case control study | 3 | 56 |
| Maio | Aesthetic Plastic Surgery | Expert Opinion / Narrative Review | 5 | 56 |
| Matarasso 1998 | Dermatologic Surgery | Expert Opinion / Narrative Review | 5 | 54 |
| Satriyasa 2019 | Clinical Cosmetic And Investigational Dermatology | Expert Opinion / Narrative Review | 5 | 53 |
| Gart 2016 | Clinics In Plastic Surgery | Expert Opinion / Narrative Review | 5 | 53 |
| Kane 2010 | Journal Of Drugs In Dermatology | Expert Opinion / Narrative Review | 5 | 53 |
| Huang 2000 | Dermatologic Surgery | Case control study | 3 | 52 |
| Carruthers 2013 | Dermatologic Surgery | Expert Opinion / Narrative Review | 5 | 51 |
| Lowe 1998 | Dermatologic Surgery | Expert Opinion / Narrative Review | 5 | 50 |
| Carruthers 2004 | Dermatologic Surgery | Randomised controlled trial | 1 | 49 |
| Carruthers 1998 | Dermatologic Surgery | Expert Opinion / Narrative Review | 5 | 47 |
| Carruthers 2013 | Dermatologic Surgery | Randomised controlled trial | 1 | 47 |
| De Boulle 2010 | Clinical Interventions In Aging | Expert Opinion / Narrative Review | 5 | 46 |
| Matarasso 2001 | Plast Reconstr Surg | Expert Opinion / Narrative Review | 5 | 44 |
| Chang 2008 | International Journal Of Dermatology | Case control study | 3 | 43 |
| Glogau 2012 | Dermatologic Surgery | Systematic review | 2 | 41 |
| Kassir 2019 | Journal Of Cosmetic Dermatology | Expert Opinion / Narrative Review | 5 | 41 |
| Carruthers 2005 | Dermatologic Clinics | Expert Opinion / Narrative Review | 5 | 40 |
| Dayan | Dermatologic Surgery | Case control study | 3 | 40 |
| Keaney | Dermatologic Surgery | Expert Opinion / Narrative Review | 5 | 40 |
| Carruthers 2013 | Dermatologic Surgery | Expert Opinion / Narrative Review | 5 | 40 |
| Carruthers 2001 | Seminars In Cutaneous Medicine And Surgery | Expert Opinion / Narrative Review | 5 | 40 |
| Grimes 2009 | Dermatologic Surgery | Randomised controlled trial | 1 | 39 |
| Min 2015 | Aesthet Surg J | Randomised controlled trial | 1 | 38 |
| Liu 2019 | J Cosmet Dermatol-Us | Expert Opinion / Narrative Review | 5 | 38 |
| Chang 2016 | Aesthetic Surgery Journal | Individual cohort study | 2 | 37 |
| Fagien 2001 | Clinics In Plastic Surgery | Expert Opinion / Narrative Review | 5 | 35 |
| Carruthers 2010 | Dermatol Surg | Randomised controlled trial | 1 | 35 |
| Cox 2003 | Dermatologic Surgery | Case control study | 3 | 35 |
| Fabi 2016 | Dermatologic Surgery | Expert Opinion / Narrative Review | 5 | 35 |
| Kapoor 2010 | Dermatologic Surgery | Randomised controlled trial | 1 | 35 |
| Niamtu 1999 | Journal Of Oral And Maxillofacial Surgery | Expert Opinion / Narrative Review | 5 | 35 |
| Schlessinger | Aesthetic Surgery Journal | Expert Opinion / Narrative Review | 5 | 34 |
| Baumann 2009 | Aesthetic Surgery Journal | Randomised controlled trial | 1 | 34 |
| Resneck 2007 | J Am Acad Dermatol | Cross sectional study | 4 | 34 |
| Le Louran 2001 | Aesthetic Plastic Surgery | Expert Opinion / Narrative Review | 5 | 33 |
| Pena 2007 | Seminars In Cutaneous Medicine And Surgery | Expert Opinion / Narrative Review | 5 | 33 |
| Dover 2018 | Dermatologic Surgery | Expert Opinion / Narrative Review | 5 | 32 |
| Rzany 2009 | Journal Of The European Academy Of Dermatology And Venereology | Expert Opinion / Narrative Review | 5 | 32 |
| Bertossi 2019 | Laser Med Sci | Case control study | 3 | 32 |
| Steinsapir 2015 | Ophthalmic Plastic And Reconstructive Surgery | Case control study | 3 | 32 |
| Sethi 2021 | Aesthetic Plastic Surgery | Expert Opinion / Narrative Review | 5 | 31 |
| Allen 2012 | Dermatologic Surgery | Randomised controlled trial | 1 | 31 |
| Flynn 2012 | Journal Of Cosmetic Dermatology | Expert Opinion / Narrative Review | 5 | 31 |
| Nestor 2017 | Aesthetic Surgery Journal | Expert Opinion / Narrative Review | 5 | 30 |
| Cheng 2007 | Clinical Interventions In Aging | Expert Opinion / Narrative Review | 5 | 30 |
| Dorizas 2013 | Dermatologic Clinics | Expert Opinion / Narrative Review | 5 | 30 |
| Sorensen 2015 | Journal Of Drugs In Dermatology | Expert Opinion / Narrative Review | 5 | 30 |
| Molina 2015 | Dermatologic Surgery | Single-arm clinical trial | 3 | 29 |
| Jabbour 2017 | Plastic And Reconstructive Surgery | Case series | 4 | 28 |
| Maas 2012 | Aesthetic Surgery Journal | Expert Opinion / Narrative Review | 5 | 27 |
| Dessy 2011 | American Journal Of Clinical Dermatology | Expert Opinion / Narrative Review | 5 | 27 |
| Carruthers 2009 | Dermatologic Clinics | Expert Opinion / Narrative Review | 5 | 27 |
| Zhu 2016 | Dermatologic Therapy | Randomised controlled trial | 1 | 27 |
| Michaels 2012 | Aesthetic Surgery Journal | Randomised controlled trial | 1 | 26 |
| de Almeida 2015 | Dermatol Surg | Case control study | 3 | 26 |
| Mahmoud 2015 | Dermatol Surg | Randomised controlled trial | 1 | 26 |
| Cather 2005 | Dermatologic Clinics | Expert Opinion / Narrative Review | 5 | 26 |
| Awaida 2018 | Plastic And Reconstructive Surgery | Expert Opinion / Narrative Review | 5 | 26 |
| Cohen 2009 | Aesthetic Surgery Journal | Prospective cohort study | 2 | 25 |
| Anido 2017 | Clinical Cosmetic And Investigational Dermatology | Expert Opinion / Narrative Review | 5 | 25 |
| Carruthers 2015 | Dermatol Surg | Case series | 4 | 25 |
| Rivers 2015 | Dermatol Surg | Randomised controlled trial | 1 | 25 |
| Brandt 2005 | Dermatologic Clinics | Expert Opinion / Narrative Review | 5 | 25 |
| Jeon 2013 | Dermatology | Prospective cohort study | 2 | 25 |
| Helmy 2018 | J Cosmet Laser Ther | Case series | 4 | 25 |
